# Novel technique for treating grass carp (*Ctenopharyngodon idella*) by combining plasma functionalized liquids and Ultrasound: Effects on bacterial inactivation and quality attributes

**DOI:** 10.1016/j.ultsonch.2021.105660

**Published:** 2021-07-03

**Authors:** Okon Johnson Esua, Jun-Hu Cheng, Da-Wen Sun

**Affiliations:** a School of Food Science and Engineering, South China University of Technology, Guangzhou 510641, China; b Academy of Contemporary Food Engineering, South China University of Technology, Guangzhou Higher Education Mega Centre, Guangzhou 510006, China; c Engineering and Technological Research Centre of Guangdong Province on Intelligent Sensing and Process Control of Cold Chain Foods, & Guangdong Province Engineering Laboratory for Intelligent Cold Chain Logistics Equipment for Agricultural Products, Guangzhou Higher Education Mega Centre, Guangzhou 510006, China; d Food Refrigeration and Computerized Food Technology (FRCFT), Agriculture and Food Science Centre, University College Dublin, National University of Ireland, Belfield, Dublin 4, Ireland

**Keywords:** Plasma functionalized water, Plasma functionalized buffer, Fish decontamination, Reactive oxygen species, Reactive nitrogen species

## Abstract

A novel technique for treating grass carp by combining plasma functionalized liquids and ultrasound to inactivate bacteria was developed. The effects of the plasma functionalized liquids (PFL) including plasma functionalized water (PFW) and buffer (PFB) and their respective combination with ultrasound treatment (USPFW and USPFB) on the oxidative and physical qualities of grass carp were also investigated. Individual applications of PFW and PFB significantly reduced the populations of *Escherichia coli* and *Shewanella putrefaciens* in the range of 0.31–1.18 log CFU/g, compared with the control with a reduction of 0.18 log CFU/g, while combined treatments of USPFW and USPFB presented additional reductions of 0.05–0.65 log CFU/g*,* with potential synergy demonstrated for PFW and ultrasound. The treatment resulted in improved biomedical index and nutritional value of fatty acids and lipids, protein structural unfolding, increased lipid oxidation and protein degradation with values within the acceptable limits, and the combined treatment was more effective for retarding the hardness reduction in grass carp, while the colour change was also significantly affected, resulting in increased whiteness. The results indicated that the combined treatments may be a promising approach to improving the quality of seafood products.

## Graphical abstract

**Figure:**
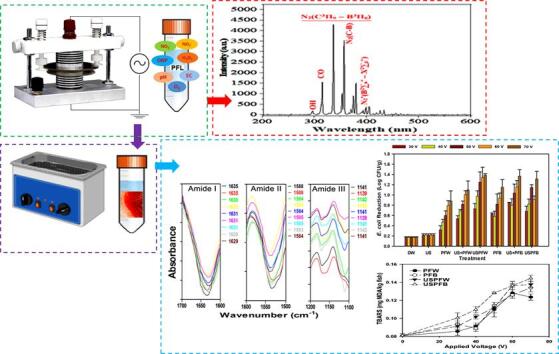

## 1 Introduction

Grass carp (*Ctenopharyngodon idella*) is the most popular freshwater fish in the world, but rapid deterioration from postmortem microbial, oxidative and biochemical changes have hindered their further utilization [1]. Various techniques have been developed for seafood products sanitization, and subjecting liquids to cold plasma discharge to produce plasma functionalized liquids (PFL) emerges as effective eco-friendly sanitisers over direct cold plasma applications [2], [3]. The use of PFL is continuously being explored for processing meat and seafood products, including shelf life extension, delay in microbial growth and quality degradation, alternative curing, and assisted thawing [4], [5], [6].

However, the use of PFL is seen as adding nitrates (NO_3_^–^) and nitrites (NO_2_^–^) to meat and seafood products and has become a challenging safety issue, since NO_2_^–^ has the potential to form carcinogenic N-nitroso compounds in these products, and the USA Food and Drug Administration approves 500 ppm of NO_3_^–^ and 200 ppm of NO_2_^–^ on edible products [2], [7]. As such, alternative approaches such as hurdle technology are necessary for improving the quality and safety of these products without the risk of increased levels of residual NO_2_^–^ from PFL application. The combination of functionalized liquids with nonthermal approaches like ultrasound, ultraviolet radiation (UV-C) and tea polyphenol have been investigated with encouraging results, and plasma functionalized water (PFW) combined with ultrasound treatment has also been explored for treating chicken meat [8], [9], [10], [11], [12].

Ultrasound treatment exhibits antimicrobial properties through the mechanism of cavitation and sonolysis, as ultrasound generates compression, mechanical shockwaves and rarefaction [3], [13], [14], and produces H atoms, H_2_O_2_, and •OH radicals that damage functional and cellular components, resulting in cell lysis. These actions also affect myofibrils and muscle structure, increase water holding capacity and prevent marinate and water losses in muscle food, thereby influencing quality [9], [10]. In addition, high-intensity ultrasound operates at frequencies of 16–100 kHz and can lead to local high pressure and temperature in the liquid media, causing damage to the structure of the cellular organelle and leading to proteolysis and lipid and protein oxidation [8], [9], [10]. Therefore, the sanitization efficacy of PFL including PFW and plasma functionalized buffer (PFB) when combined with ultrasound technology is expected to be improved as their simultaneous application can facilitate the actions of reactive species in PFL due to micro-cracks produced in bacterial cell membranes by ultrasound treatment. This will not only allow for the optimisation of microbial inactivation but can also preserve the inherent sensory and nutritional properties of seafood products.

To date, the combination of PFL and ultrasound treatment as a hurdle technology has not been explored for seafood products decontamination. Therefore, the objective of this study was to explore the inactivation of *Escherichia coli* (*E. coli*) and *Shewanella putrefaciens* (*S*. *putrefaciens*) inoculated on grass carp by the application of PFW and PFB and their combination with ultrasound treatment, and evaluate their effects on some quality characteristics of grass carp.

## 2 Materials and methods

### 2.1 Bacteria strains and inoculum preparations

*E. coli* ATCC25922 and *S*. *putrefaciens* ATCC BAA-1097 were obtained from Guangzhou Microbial Culture Centre (Guangzhou, China) and stored at −80 ℃. Tryptic soy-yeast extract broth (TSB-YE) and Luria-Bertani (LB) broth were adopted for activating the strains of *E. coli* and *S*. *putrefaciens*, respectively, and retained as frozen stocks in sterile tubes with glycerol at −80 ℃. The frozen strains of *E. coli* were plated on tryptic soy agar, while the strains of *S*. *putrefaciens* were plated on nutrient agar and incubated overnight at 37 ℃ for 18–24 h to obtain pure single colonies. Subsequently, the loopfuls of isolated colonies were suspended in TSB-YE and LB broth, respectively for *E. coli* and *S*. *putrefaciens*, and incubated at 37 ℃ until the required optical density of 0.5 – 0.6 was achieved at 600 nm. Bacteria cells were harvested by centrifuging (JW-3024HR, Anhui Jiaven Equipment Industry Co., Ltd., Hefei, China) at 3000 × g and 4 ℃ for 15 min to obtain pellets, which were then washed twice and re-suspended in sterile 0.85% NaCl solution with final inoculum concentrations of 10^6^–10^7^ CFU/mL.

### 2.2 Sample collection and preparation

Fresh farmed grass carp (18–24 cm and 1.8–2.0 kg) were filleted and purchased from a local supermarket (Guangzhou, China), which were conveyed in ice to the laboratory within 1 h for storage at −20 ℃. The fillets were cut into cuboids of 4.0 x 2.0 x 2.0 cm with 10 ± 0.5 g in weight, which were spread on sterile aluminium foil placed in a biosafety cabinet (BSC-1100IIB2-X, Jinan Biobase Biotech Co., Ltd., Jinan, China). The cuboids were individually inoculated by gently spreading 100 µL of each inoculum of *E. coli* and *S*. *putrefaciens* around the entire surface area, which were air-dried in the biosafety cabinet for 1 h at room temperature of 25 ℃ to allow for bacteria attachment on the cuboids. The final concentrations of both pathogens inoculated on the cuboids were in the range of 5–6 log CFU/g, which were then used as samples in the subsequent experiments.

### 2.3 Equipment set-up and configuration

For the current study, a dielectric barrier discharge (DBD) system (CTP-2000 K, Nanjing Suman Electronics Co., Ltd., Nanjing, China) was used to generate cold plasma, and the detailed description of the equipment can be found in previous studies [15], [16]. The plasma reactor consisted mainly of low and high voltage electrodes separated by a circular upper quartz glass, which served as the dielectric barrier and was operated with air as the feed gas.

The ultrasound treatment was achieved using a bath-type sonochemical reactor (SB25-12D, Ningbo Xinzhi Ultrasonic Equipment Co., Ltd., Ningbo, China) at a frequency of 40 kHz and an acoustic power level of 500 W. The inner tank of the ultrasonic bath had dimensions of 500 x 300 x 150 mm (L x W x H) and a volume of 22.5 L, thus the volumetric power of the reactor was determined by the colourimetric method as 17.45 W/L using equations below:

(1) $$P_{\mathrm{diss}}=mC_{p}\left(\frac{\mathrm{dT}}{\mathrm{dt}}\right)$$

(2) $$Volumetricpower=\frac{P_{\mathrm{diss}}}{V}$$

where $P_{\mathrm{diss}}$ is ultrasonic power dissipated into the water in the tank, m is mass of the water in the tank (kg), $C_{p}$ is the specific heat capacity of water (4187 J/kg K), (dT/dt) is the slope of the temperature versus time curve, and V is the volume of water in the tank (L).

### 2.4 Generation of PFL and decontamination treatments

**Fig. 1:**
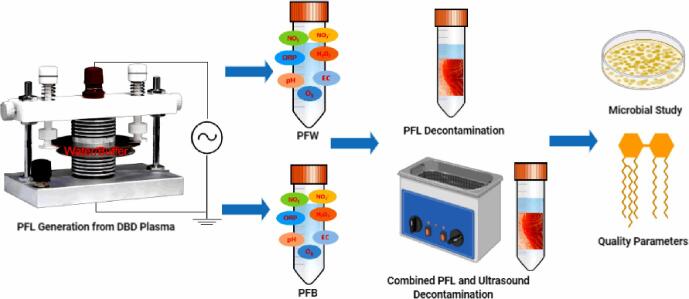
Schematic representation of the experimental procedure.

The PFL was generated by exposing 20 mL of the liquids in a petri dish to the cold plasma between the high and low voltage electrodes. A schematic representation of the experimental process is given in Fig. 1. Deionized water (DW) was used to prepare PFW, while the citrate–phosphate buffer was prepared by mixing 4.29 g of Na_2_HPO_4_ and 11.01 g of C_6_H_8_O_7_ to a volume of 500 mL, which was then used to prepare PFB. The DBD plasma system was operated at an input voltage of 30, 40, 50, 60, and 70 V and the duration of plasma exposure to the liquids was 8 min. During plasma discharge, a distance of 5 mm was maintained between the liquid surface and the high voltage electrode, which was separated by the dielectric barrier. The PFW and PFB generated were transferred to sterile falcon tubes and cooled for 2 min to void the temperature effect during decontamination, and the basic physicochemical properties were characterized according to the methods described in our previous studies [15]. For individual PFL treatment, decontamination was achieved by immersing the samples in the sterile falcon tubes containing either PFW or PFB, which was placed on an orbital shaker (WSZ-10A, Shanghai Yiheng Technology Co., Ltd., Shanghai, China) operating at 150 rpm for 4 min at room temperature of 25 ℃.

For the combined treatment of PFL and ultrasound, the samples were immersed in the sterile falcon tubes containing either PFW or PFB, and the tube was fixed on a tube rack at the centre of the ultrasound bath and sonicated for 4 min at room temperature of 25 ℃, which was identified as either USPFW or USPFB. For comparison, samples were immersed in DW (corresponding to 0 V) and placed on the orbital shaker operating at 150 rpm for 4 min without PFL and ultrasound treatment were used as control. Separate rounds of experiments were performed for microbial decontamination, firmness and colour properties, lipid oxidation profile and the degradation of nitrogenous compounds, respectively, and each round of experiments was repeated twice. Samples were also subjected to ultrasound treatment alone for 4 min, designated as US during microbial decontamination and the effect of combined decontamination was evaluated according to the equation below:

(3) $$\mathrm{Effect}=Y_{\mathrm{ij}}-\left(Y_{i}+Y_{j}\right)$$

where Y is microbial log reduction, i and j are the individual treatment of ultrasound and PFL, respectively. The synergistic or antagonistic effect is established by positive or negative values, respectively [17].

The residual nitrite and nitrate of samples after decontamination were also evaluated by homogenizing 5 g of samples and extracted with hot water according to the method of the Association of Official Analytical Chemists (AOAC) as modified by Mohamed et al. [18]. Thereafter, the nitrite and nitrate contents were determined as described in our previous study [15] and expressed as mg/kg of fish.

### 2.5 Microbiological characterization

Microbiological characterization was performed as described in a previous study [15] using Sorbitol McConkey agar and plate count agar for *E. coli* and *S*. *putrefaciens*, respectively. Bacteria colonies were enumerated and expressed as log CFU/g following incubation of the agar plates at 37 ℃ for 24 h.

### 2.6 Lipid oxidation profile

#### 2.6.1 Peroxide value (PV)

The PV of the samples were determined by the method of Gokoglu et al. [19] with slight modifications. Briefly, 5 g of samples were minced and mixed with 5 mL chloroform and 7.5 mL glacial acetic acid and filtered. Freshly prepared saturated potassium iodide solution was added to the filtrate intermittently in drops (1 mL) and incubated in the dark for 5 min at a room temperature of 25 °C with occasional shaking. The iodine content of the mixture was released by adding 50 mL of DW and the solution was titrated with 0.01 N sodium thiosulphate until the yellow colour disappeared. Thereafter, 0.5 mL of 1% starch solution was added to the mixture and titration continued until the blue colour disappeared. The PV value was expressed in mEq peroxide/kg of fish by

(4) $$PV=\frac{\left(S-B\right)*N}{m}*1000$$

where Sand Bare the volumes (mL) of the titrant for samples and blank during titration, respectively, N is the normality of Na_2_S_2_O_3_ (mEq/mL), 1000 is the conversion of units (g/kg), and m is the mass of the sample (g).

#### 2.6.2 Thiobarbituric acid reactive substances (TBARS)

The TBARS value of the samples was obtained with the method of Cheng et al. [20]. An amount of 5 g of the sample was homogenized in 20 mL of DW, mixed with 25 mL of 20% trichloroacetic acid and centrifuged for 10 min at 8000 rpm. The filtrate was diluted with double distilled water to 50 mL, and 10 mL of the diluent was mixed with 10 mL of 0.06 M thiobarbituric acid and heated to 100 ℃ for 15 min in a water bath (HH-501, Changzhou Aohua Instrument Co., Ltd., Changzhou, China) for developing a pink colour. The absorbance of the mixture was measured at 532 nm using a UV spectrophotometer (UV-1800, Shimadzu Co., Kyoto, Japan) after cooling with running water for 5 min. The TBARS values were expressed as the amount of malondialdehyde (MDA) in the sample using the equation below:

(5) $$TBARS\left(mg\frac{MDA}{kg}\right)=\frac{\mathrm{Abs}*MW*DF}{\varepsilon *l}*1000$$

where Abs is the absorbance of samples, MW is the molecular weight of MDA (72.063 g/mol), DF is dilution factor, ε is the molar extinction coefficient of MDA (1.56*10^5^ L/mol cm), 1000 is the conversion factor of units (mg/g), and l is path length (cm).

#### 2.6.3 Para-anisidine value (AnV)

The AnV of the samples were determined according to the method of Gokoglu et al. [19] with slight modifications. An amount of 5 g of the sample was minced and mixed with 25 mL of n-hexane and filtered. Thereafter, 2.5 mL of the filtrate was mixed with 1 mL of 0.5% *para*-anisidine solution (2.5 g/L in glacial acetic acid) and incubated for 10 min at room temperature of 25 ℃. The absorbance of the mixture was read at 350 nm and the AnV was calculated by

(6) $$AnV=\frac{\left[25*\left\{1.2A_{2}-A_{1}\right\}\right]}{m}$$

where $A_{1}$ and $A_{2}$ are the absorbances before and after adding *para*-anisidine to the filtrate, respectively, and m is the mass of the sample (g).

### 2.7 Determination of total volatile basic-nitrogen (TVB-N)

The TVB-N of the samples was determined from the steam distillation method reported in Cheng et al. [20] with slight modifications. An amount of 5 g of the sample was chopped into fine pieces, mixed with 45 mL of 0.6 M perchloric acid and centrifuged at 3000 rpm for 10 min. The ensuing filtrate was mixed with 50 mL of 40% NaOH and distilled in a Kjeltec distillation unit (8100, FOSS Tecator, Hillerod, Denmark) for 5 min using 50 mL of DW as control. The distillate obtained was channelled into a conical flask containing 50 mL of 40 g/L boric acids, which was then mixed with a combined indicator of 0.1 g bromocresol green and 0.1 g methyl red in 100 mL of 93% ethanol. Thereafter, the solution was titrated with 0.01 M HCl until a faint pink colour appeared. The TVB-N values of the samples were expressed as mg N/100 g according to the equation below:

(7) $$TVB-N=\frac{\left(S-B\right)*C*14}{m}*100$$

where Sand Bare the volumes (mL) of the titrant for the samples and blank during titration, respectively,

C is the concentration of HCl (M) and m is the mass of the sample (g).

### 2.8 Fatty acid profile analysis

Fatty acid composition was determined as fatty acid methyl esters (FAMEs) from a gas chromatography system equipped with flame ionization detector (Agilent 7890A, Santa Clara, CA, USA) according to the method of Shiekh and Benjakul [21] with modifications. Grass carp was extracted with water:isopropanol:cyclohexane (11:8:10) mixture and rotoevaporated to obtain fish oil, and the FAMEs were obtained by transesterification of the fish oil with 0.5 N methanolic potassium hydroxide and 0.5 N methanolic boron trifluoride and subsequent extraction with hexane and sodium chloride. Peaks were identified based on the retention time of reference standards and the relative content of each fatty acid was evaluated by the area normalization method according to the following equation:

(8) $$\mathrm{FAXarea}\%=\frac{\mathrm{AX}}{\mathrm{AR}}x100$$

where FAX is the fatty acid to be quantified, AX is area of methyl ester X, and AR is the total area of the chromatogram.

### 2.9 Fourier transform infrared (FTIR) spectroscopy

FTIR spectral measurements of fillets blocks (0.5 x 0.5 x 0.2 cm) placed in direct contact with the ATR ZnSe crystal were taken between 4000 and 400 cm^−1^ at room temperature of 25 ℃ from a NICOLET iS50 FTIR spectrometer (Thermo Electron Inc., San Jose, CA, USA) according to Ovissipour et al. [22]. Thirty-two scans collected along different locations of each fillet surface at a resolution of 4 cm^−1^ were averaged and evaluated using the OMNIC spectra software.

### 2.10 Textural profile analysis (TPA)

The TPA was evaluated at two consecutive strain cycles of 15% from a texture analyzer (TA.XTplusC, Stable Micro Systems Ltd., Surrey, UK) equipped with a 50 mm cylindrical (P/50) aluminium probe as previously described by Feng et al. [12]. The test speed was set at 5 mm/s at a triggering force of 0.05 N, while the pre-test and post-test speeds were set at 2 and 5 mm/s, respectively [23], [24]. The hardness, adhesiveness, springiness, cohesiveness and chewiness of the samples were obtained from the force–time profile using Version 7.0.6.0 of the proprietary Exponent Connect software (Stable Micro Systems Ltd., Surrey, UK).

### 2.11 Colour measurements

The lightness (L*), redness (a*) and yellowness (b*) of the samples were measured along six symmetrical sections using a chromameter (CR-400, Konica Minolta Inc., Osaka, Japan) according to the CIELAB coordinates [12]. The chroma meter was standardised with a white calibration tile before each measurement. The average values of L*, a*, b* coordinates from the six sections were documented and the colour parameters were expressed as net colour difference (NCD) and whiteness index (WI) using the equations below:

(9) $$\mathrm{NCD}=\sqrt{{\left(L_{o}^{*}-L^{*}\right)}^{2}+{\left(a_{o}^{*}-a^{*}\right)}^{2}+{\left(b_{o}^{*}-b^{*}\right)}^{2}}$$

(10) $$\mathrm{WI}=\sqrt{\left[{\left(100-L^{*}\right)}^{2}+{a^{*}}^{2}+{b^{*}}^{2}\right]}$$

where $L_{0}^{*}$, $a_{0}^{*}$ and $b_{0}^{*}$are the initial values of $L^{*},a^{*}$ and $b^{*}$.

### 2.12 Statistical analysis

SigmaPlot 12.0 software (Systat Software Inc., CA, USA) was adopted for performing statistical analysis from triplicate samples in a completely randomized block design and data were expressed as mean ± standard error of measurement (SEM). The effects of treatment were evaluated by the two-way analysis of variance (ANOVA) at *p* < 0.05 from the Holm-Sidak test, typically recommended as the first-line procedure for most multiple comparison testing, and principal component analysis (PCA) was performed from The Unscrambler X 10.4 (CAMO Software AS, Oslo, Norway).

## 3 Results and discussion

### 3.1 Changes in physicochemical properties of PFL and residual nitrite and nitrate contents

**Fig. 2:**
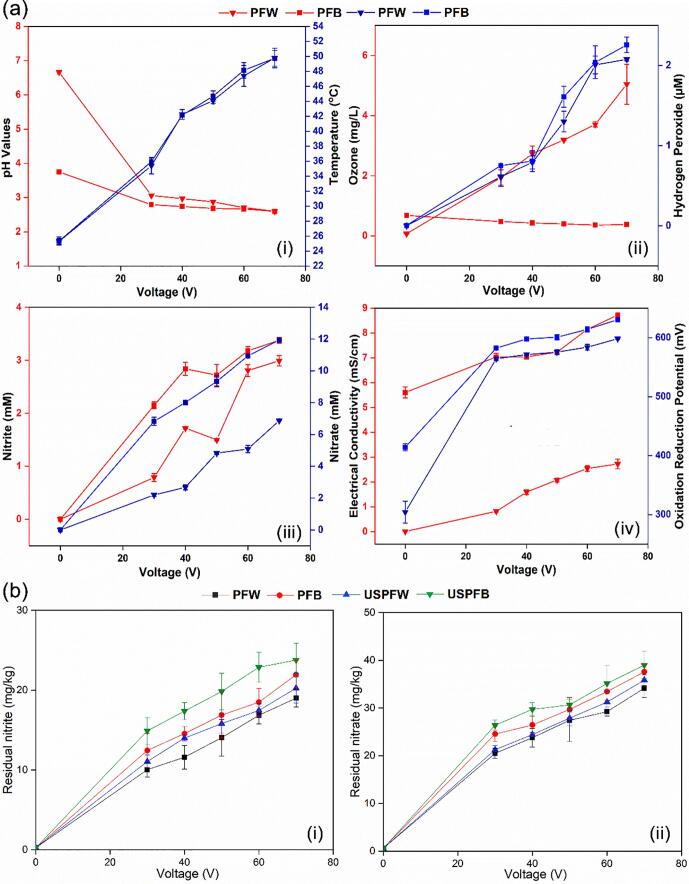
(a) Physicochemical properties of PFW and PFB during liquids exposure to cold plasma: (i) pH and temperature, (ii) ozone and hydrogen peroxide, (iii) nitrites and nitrates, (iv) EC and ORP, and (b) residual (i) nitrite and (ii) nitrate content of grass carp during decontamination.

The typical emission spectra of the DBD plasma system used in the current study has been described in our previous studies [15]. It was reported that excited atomic nitrogen and oxygen species dominated, which produced acidic species like nitric acid (HNO_3_), peroxynitrous acid (ONOOH) and nitrous acid (HNO_2_) in DW and buffer solution at the gas–liquid interphase, resulting in increased acidification with increasing voltage. The acidification was more drastic for PFW reaching 2.60 from an initial pH of 6.67, compared with 2.59 for PFB from an initial value of 3.75, while the temperature increase was similar for both solutions and reached 49.8℃ after 70 V (Fig. 2a). The O_3_ content of PFW showed an increasing trend with increasing voltages and reached 5.05 mg/L, while PFB exhibited a decreasing trend and reached 0.38 mg/L from an initial value of 0.68 mg/L. Both PFW and PFB showed a similar increasing trend and values for H_2_O_2_ contents and reached a maximum value of 2.26 µM·H_2_O_2_ exhibits strong oxidative potentials especially in an acidic environment, while O_3_ is typically a long-lived specie that influences chemical reactions and contributes to the antibacterial potential of PFL [15], [25], [26], [27], [28], [29].

The NO_2_^–^ and NO_3_^–^ contents of both solutions exhibited an increasing trend with increasing voltage application, with PFB presenting higher values. The values reached 2.99 and 3.38 mM for NO_2_^–^ and 6.87 and 11.94 mM for NO_3_^–^ for PFW and PFB, respectively. The increases in NO_2_^–^ and NO_3_^–^ could be linked to the dissolving oxides of N_2_ that were generated during the gas-phase reactions of H_2_O, N_2_ and O_2_ molecules [30], [31], [32], while the higher values of NO_3_^–^ might be explained by the transformation of NO_2_^–^ to NO_3_^–^, especially in the acidic environment as a result of the unstable nature of NO_2_^–^. Similar trends were observed for the electrical conductivity (EC) and oxidation–reduction potential (ORP) of both solutions, with values ranging from 0.82 to 2.73 mS/cm and 7.04–8.72 mS/cm for EC and 564.50–598.43 mV and 582.33–630.79 mV for ORP for PFW and PFB, respectively. The EC is associated with the nature of charged species and excited ions, while the ORP shows the concentration and strength of oxidizers in PFL and can change the redox state of microbes, which are essential to the antibacterial potential of PFL [28], [33].

The residual NO_2_^–^ and NO_3_^–^ the content of fillets were also evaluated and results showed increases with increasing voltage applications (Fig. 2b) to reach 19.01, 21.94, 20.26, and 23.77 mg/kg for nitrite, and 34.17, 37.60, 35.87, and 39.01 mg/kg for nitrate, respectively for PFW, PFB, USPFW and USPFB. The combined treatment presented slightly higher values probably due to ultrasound-induced pyrolysis reaction, but values were within the maximum residue levels of 50–175 mg/kg for NO_2_^–^ and 10–250 mg/kg for NO_3_^–^ [34].

### 3.2 Changes in the reduction of pathogenic bacteria

**Fig. 3:**
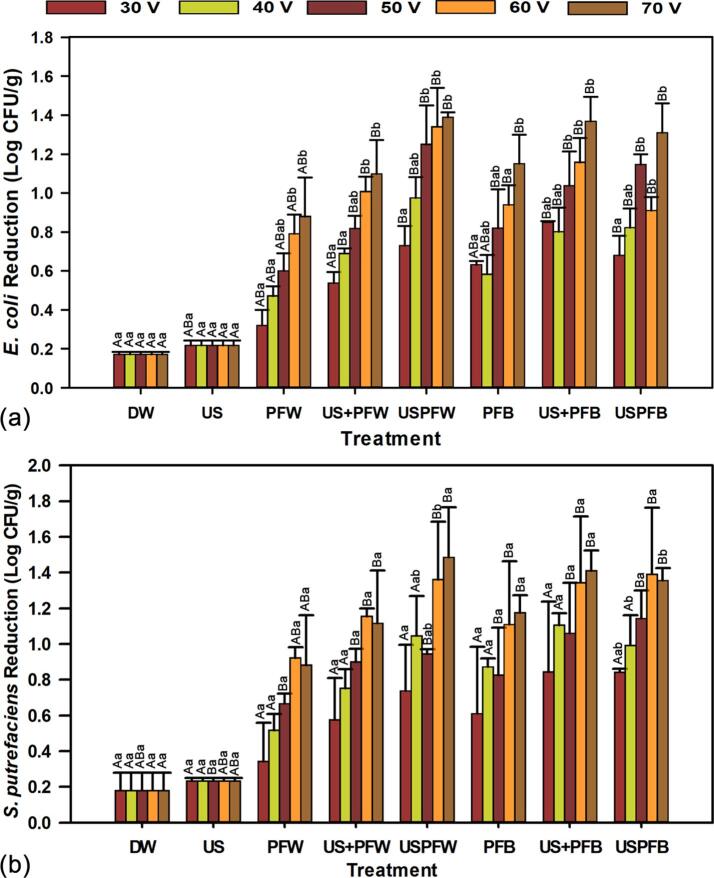
Effects of decontamination methods on the inactivation of (a) *E. coli* and (b) *S*. *putrefaciens* inoculated on grass carp. Error bars represent standard error of measurement. Different lowercase letters are significantly different (*p* < 0.05) within voltage application, and different uppercase letters are significantly different within DW, US, PFW, US + PFW, USPFW, PFB, US + PFB, and USPFB.

The effectiveness of inactivating *E. coli* and *S*. *putrefaciens* inoculated on grass carp is presented in Fig. 3. Immersing the samples in DW reduced the initial counts of *E. coli* and *S*. *putrefaciens* by 0.17 and 0.18 log CFU/g, respectively, while ultrasound treatment alone (US) led to reductions of 0.22 and 0.23 log CFU/g, respectively. Significant reductions (*p* < 0.05) in the population of pathogenic bacteria were observed for individual treatments of PFL, especially for high voltage application, presenting the highest reductions of 0.88 and 0.92 log CFU/g for PFW, and 1.15 and 1.18 log CFU/g for PFB, respectively for *E. coli* and *S*. *putrefaciens*. The combination of PFL and ultrasound were more effective in reducing the initial counts of both pathogens on grass carp with significant reductions at all the voltage levels for *E. coli*, while the reductions in *S*. *putrefaciens* as a result of combined treatment were only significant for the voltage range of 50–70 V. The highest reductions as a result of combined treatment were 1.39 and 1.49 log CFU/g for USPFW and 1.31 and 1.39 log CFU/g for USPFB, respectively for *E. coli* and *S*. *putrefaciens*.

The observed bacterial reductions can be attributed to the strong acidification induced during plasma-liquid interaction, and the generation of NO_2_^–^ and H_2_O_2_ progresses to produce peroxynitrous acid (ONOOH) and peroxynitrite anion (ONOO^–^), which exhibits high reactivity and oxidation potential with biological materials [2], [35], [36]. Moreso, the resistance of bacteria is weakened in an acidified environment, which ensures improved penetration of reactive oxygen and nitrogen species (RONS) into the cell walls of bacteria, compromising cell membrane integrity [37]. The results indicated that immersing the samples in PFB produced better reductions in both pathogens when compared with PFW, probably due to the formation of more RONS as well as increased ORP and EC in PFB, which may have resulted in the inactivation of more bacteria cells.

The use of ultrasound as an additional hurdle technology has been reported to significantly increase the ORP of functionalized liquids and increase antibacterial efficiency [38]. This may be due to the production of free radicals from the thermal dissociation of the solution by cavitation-accompanied localized pressure spots and high temperature. Besides, bubble implosion during cavitation generates a great amount of energy, which increases the rupture of the cytoplasmic membrane, making the bacteria in the current study more susceptible to RONS from PFL [9], and suggest the possible synergistic antibacterial effect of the combination of PFL and ultrasound. However, the synergy was more effective with PFW, which produced additional log reductions in the range of 0.30–0.65 as compared with 0.05–0.33 for PFB.

In addition, the results also illustrated that log reductions from simultaneous treatment (USPFW) were greater than the sum of the log reductions from individual treatments (US + PFW) for both pathogens, showing synergistic effects of 0.20, 0.29, 0.43, 0.33 and 0.30 log CFU/g for *E. coli*, and 0.16, 0.30, 0.05, 0.21 and 0.37 log CFU/g for *S*. *putrefaciens*, respectively at 30, 40, 50, 60 and 70 V. On the other hand, log reductions from USPFB were only greater than the log reductions from US + PFB at 40 and 50 V for *E. coli*, and at 50 and 60 V for *S*. *putrefaciens,* suggesting less synergistic and more antagonistic effects. Furthermore, paired analysis between USPFW and US + PFW presented significant differences (*p* < 0.05) at all voltage levels for *E. coli* and at 60 and 70 V for *S*. *putrefaciens*, further confirming synergistic effect, while paired analysis between USPFB and US + PFB only presented significant differences at 50 V for *E. coli* and at 50 and 60 V for *S*. *putrefaciens*, further confirming less synergistic and more antagonistic effects for this treatment. Similar synergistic and antagonistic effects have been recorded during ultrasound combination with aqueous ozone and peroxyacetic acid [17], [39].

Although there are no studies on using the combination of PFL and ultrasound to decontaminate seafood products, Royintarat et al. [10] observed that the synergistic effect of PFW and ultrasound on chicken meat produced 1.12 and 0.86 log CFU/mL reductions in *E. coli* and *Staphylococcus aureus*, respectively, compared with 0.56 and 0.43 log CFU/mL obtained when PFW was applied alone. Similar trends and results have also been reported for *E. coli*, *V. parahaemolyticus, L. monocytogenes* and *Staphylococcus* spp. on sliced shad, salmon fillets and chicken breast when EFW was combined with ultrasound treatments [8], [9], [11].

### 3.3 Changes in lipid oxidation profile

#### 3.3.1 PV contents

**Fig. 4:**
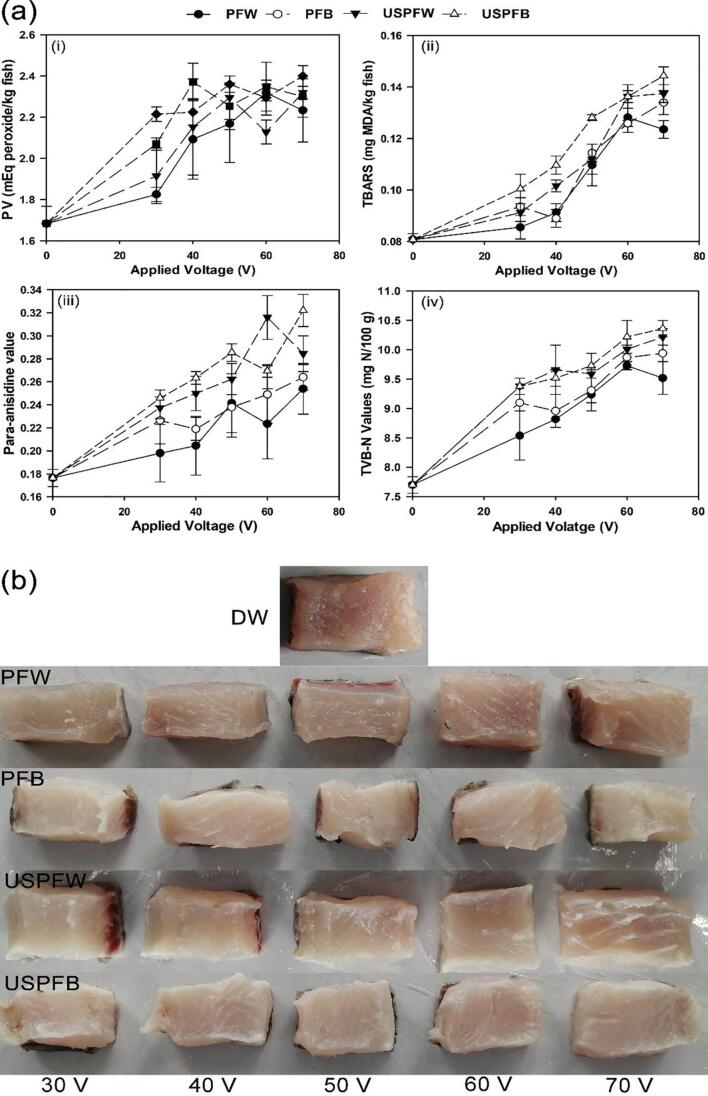
(a) Effects of decontamination methods on quality profile: (i) PV, (ii) TBARS and (iii) AnV, (iv) TVB-N and (b) visual colour of grass crap.

The change in the PV of the samples is presented in Fig. 4a(i). Immersing the samples in PFW and PFB and their combinations with ultrasound showed significant increases (p < 0.05) in the PV with increasing the applied voltages as compared with immersing in DW. The increases were especially significant for higher voltages. Compared with 1.68 mEq peroxide/kg fish for immersing in DW, the highest PV values of 2.319, 2.320, 2.349, and 2.400 mEq peroxide/kg fish were obtained for PFW, PFB, USPFW and USPFB, respectively.

The current results are comparable to that for fresh mackerel decontaminated with DBD plasma as reported by Albertos et al. [40], who observed a significant increase in PV with increasing voltage and treatment time from 6.89 to 35.57 mEq O_2_/kg lipids, ascribed to the strong oxidative capacities of reactive species generated by DBD plasma. Zhang et al. [41] also reported an increase in PV from an initial value of 0.127 to 0.177 and 0.768% for immersing bighead carp in OFW and ozone gas-floatation in DW, respectively. In contrast, applications of microwave produced no significant effect on the PV of grass carp [42] or a reduction in the value [43].

Lipid oxidation is an undesirable reaction in foods, which occur through the free radical chain mechanism [44], and PV defines the initial stage of oxidative changes in fats and oils and measures the concentration of hydroperoxides and peroxides [41], [45]. On the other hand, fish oil is rich in n-3 polyunsaturated fatty acids, which is unstable and can readily oxidize to form toxic products. By immersing samples in PFW, PFB and their combination with ultrasound treatment, lipid oxidation may be encouraged due to the abundance of free radicals in PFL, as the current data indicated that immersing grass carp in PFW and PFB and their combination with ultrasound treatment could slightly increase the PV of grass carp, and the values were significantly lower than the acceptable range of 5 – 8 mEq/kg observed for the freshness of fishery products [46]. Thus, research efforts are geared towards improving the chemical composition of fish oils due to increasing pharmacological and nutritional interest [41].

#### 3.3.2 TBARS values

The lipid oxidation profile of grass carp was also evaluated using TBARS values, which is presented in Fig. 4a(ii). Results indicated that the TBARS values of the samples immersed in PFW and PFB increased slightly from an initial value of 0.077 mg MDA/kg fish with increasing applied voltages. Compared with 0.081 mg MDA/kg fish obtained for samples immersed in DW, the values were significantly (p < 0.05) affected by the individual application of PFW and PFB for all voltage levels except 30 V, reaching the highest values of 0.128 and 0.134 mg MDA/kg fish for PFW and PFB, respectively. The lower TBARS values for the samples immersed in DW suggested that only a small fraction of malondialdehyde and peroxides might have been dislodged by immersing grass carp in DW. Furthermore, the TBARS values for samples immersed in PFB were slightly higher than those in PFW, which could be attributed to the formation of more RONS in PFB. Similar trends were observed for samples immersed in PFW and PFB and subjected to ultrasound treatment for all voltage levels. The values were slightly higher than the values for the individual application, reaching 0.138 mg MDA/kg fish for USPFW and 0.144 mg MDA/kg fish for USPFB.

The observed increases in TBARS values were probably due to the oxidizing ability of PFL and ultrasound treatment. TBARS values are the secondary indicator of lipid oxidation, which is an intricate mechanism involving oxygen molecules and unsaturated fatty acids, and the generation of RONS that is a component of PFL and ultrasound treatment has been reported as a major pathway for lipid oxidation [47]. The current results are consistent with reports of Cichoski et al. [9], who immersed chicken meat in EFW and combined with ultrasound treatment. An increase in TBARS values of pork and beef treated by DBD plasma has also been reported [48]. In contrast, Albertos et al. [40] reported no significant difference (p < 0.05) between the TBARS values of mackerel fillets treated by DBD plasma and the control, while Royintarat et al. [10] also observed no significant difference in the lipid oxidation profile of the samples immersed in PFW and combined with ultrasound treatment and the control.

A TBARS level of 8 mg MDA/kg is usually considered as the limit of acceptability in fish, and it has been reported that rancidity is apparent at values greater than 3 mg MDA/kg, while values above 0.5 mg MDA/kg were detected as off-flavour by an organoleptic panel [40], [49], [50]. The current data suggest that immersing grass carp in PFW and PFB and their combination with ultrasound treatment could only minimally affect the TBARS values of the fish, and the values were within the acceptable limit.

#### 3.3.3 AnV contents

Fig. 4a(iii) summarizes the AnV contents of grass carp immersed in PFW, PFB and their combination with ultrasound treatment, and the results indicated significant increases in AnV contents with increasing applied voltages except for 30 V. The initial AnV was 0.164 and immersing in DW produced a value of 0.177, while samples immersed in PFW and PFB generated at 30 V presented values of 0.198 and 0.227, respectively, and the increase in the values continued with increasing voltage application, reaching 0.254 and 0.264 respectively at 70 V. In addition, immersing the samples in PFW or PFB and simultaneously treated by ultrasound presented higher AnV contents than without combining ultrasound treatment, reaching the highest values of 0.316 and 0.322 for USPFW and USPFB, respectively.

The increase in AnV contents indicates the apparent decomposition of primary lipid oxidation products of hydroperoxides into secondary lipid oxidation products of aldehydes and non-volatile carbonyl compounds [19], [43]. Hydroperoxides are unstable and may decompose to a variety of chemical products such as ketones, aldehydes, polymers, triglyceride dimers and cyclic compounds [42]. The current results were in agreement with other studies, in which microwave technology has been reported to significantly increase the AnV contents of grass carp [42], [43]. Nevertheless, Gokoglu et al. [19] are of the opinion that the AnV contents of quality oil should not exceed 2, suggesting that the current results are within the acceptable limit.

### 3.4 Changes in TVB-N

The effects of decontamination on the TVB-N of grass carp are presented in Fig. 4a(iv), and the results showed a significant increase (p < 0.05) in TVB-N values with increasing applied voltage for individual PFL applications. The TVB-N value increased from an initial value of 7.35 to 7.70 mg N/100 g after immersing in DW, while the values increased from 8.54 to 9.73 mg N/100 g for immersing the samples in PFW, and from 9.10 to 9.94 mg N/100 g in PFB for increasing the applied voltage from 30 to 70 V, respectively. Immersing the samples in PFW produced a better inhibiting effect on TVB-N as compared with PFB for all voltage levels. Furthermore, the TVB-N levels for samples immersed in PFW or PFB and simultaneously treated by ultrasound were slightly higher than those without ultrasound treatment, reaching the levels from 9.38 to 10.22 and 10.36 mg N/100 g for USPFW and USPFB, respectively.

TVB-N is a measure of protein degradation and the increase in the current study was attributed to the accumulation of trimethylamine, dimethylamine and ammonia from the degradation of nitrogenous compounds necessitated by the generation of free radicals in PFL by cold plasma and by ultrasound in water. Free radicals are known to directly or indirectly react with proteins and nucleic acids, triggering protein oxidation [45], [48], [51].

The majority of studies on TVB-N of fish are conducted during storage, and no reports are available on changes in TVB-N values of fish as affected by immersing in PFL for proper comparison. However, it was reported that the TVB-N values of dried Alaska pollock shreds and semi-dried raw Pacific saury decreased with increasing the treatment time of corona discharge plasma jet exposure, with levels ranging from 15.68 to 19.04 and from 17.99 to 24.74 mg%, respectively [51], [52]. In contrast, Olatunde et al. [53] found no significant differences in TVB-N values between untreated Asian sea bass and those packaged in polyethene bags and exposed to DBD plasma and reported values of 6.63–6.71 mg N/100 g for the treated samples. Chen et al. [54] also reported similar TVB-N values of 8.48–8.77 mg N/100 g for chub mackerel exposed to DBD plasma at 60 kV for 60 s.

TVB-N is frequently recognized as an index for measuring the freshness of fish and according to the Chinese standard GB 2733-2005 [55], the TVB-N rejection limit for freshwater fish is 30 mg N/100 g, while Sun et al. [45] claimed that a value of 15 mg N/100 g was an appropriate and acceptable limit of TVB-N in grass carp. Based on these levels of acceptability, it can be inferred that immersing grass carp in PFW and PFB and their combinations with ultrasound treatment could only minimally affect the TVB-N values, which were well within the acceptable limit.

### 3.5 Changes in fatty acid profile

The fatty acid composition of grass carp muscle from the lowest (30 V) and highest (70 V) voltage application for the different treatment is presented in Table 1, since variations between voltages were insignificant. Majority of the fatty acids varied significantly (*p* < 0.05) after treatment when compared with control samples, and exhibited notable similarities with relatively high content of monounsaturated fatty acids (MUFA), predominantly C18:1n9c and moderate contents of saturated fatty acids (SFA), predominantly C16:0, and polyunsaturated fatty acids (PUFA), predominantly C18:2n6c, which is in agreement with studies reported for king salmon and Pacific white shrimp [21], [56]. In general, treatment led to slight but insignificant increases and decreases in SFA, MUFA, and PUFA, with contents in the range of 24.28–32.65, 44.40–48.87, and 22.46–30.48%, relative to control sample contents of 28.51, 44.40, and 27.09%, respectively. The decreases in MUFA and PUFA may be attributed to the oxidizing ability of PFL and ultrasound treatment, confirmed by increased TBARS values. Unsaturated fatty acids are susceptible to oxidation in the presence of free radicals including ozone from PFL and cavitation-induced thermal dissociation of the solution, and the more the double bonds in a fatty acid, the more the susceptibility to oxidation [44], [57]. Similar increases and decreases have been reported for grass carp during microwave treatment, Asian sea bass, Pacific white shrimp and fresh mackerel treated with DBD cold plasma [21], [40], [43], [44], [57].

**Table 1:** Effects of decontamination on the fatty acid composition (% of total fatty acids) of grass carp.

Fatty acid	DW	PFW		PFB		USPFW		USPFB	
		30 V	70 V	30 V	70 V	30 V	70 V	30 V	70 V
C12:0	2.14 ± 0.03^a^	2.24 ± 0.01^a^	1.75 ± 0.07^b^	2.08 ± 0.04^a^	2.01 ± 0.06^ab^	1.95 ± 0.02^ab^	2.15 ± 0.01^a^	2.10 ± 0.05^a^	1.29 ± 0.08^bc^
C14:0	0.96 ± 0.08^a^	1.91 ± 0.03^ab^	1.13 ± 0.13^a^	1.81 ± 0.09^ab^	0.43 ± 0.02^ab^	1.52 ± 0.14^ab^	1.87 ± 0.08^ab^	4.79 ± 0.11^b^	0.92 ± 0.02^ab^
C15:0	1.73 ± 0.10^a^	0.68 ± 0.09^ac^	1.73 ± 0.18^ab^	0.53 ± 0.07^ac^	1.75 ± 0.11^ab^	2.60 ± 0.07^c^	1.63 ± 0.18^a^	3.89 ± 0.19^b^	2.55 ± 0.16^c^
C16:0	19.53 ± 1.08^a^	18.39 ± 0.69^a^	18.94 ± 0.80^a^	18.59 ± 0.21^a^	17.89 ± 1.18^a^	16.84 ± 0.33^a^	19.53 ± 1.43^a^	15.09 ± 2.03^a^	17.75 ± 1.67^a^
C17:0	3.93 ± 0.16^a^	4.66 ± 0.12^ab^	4.90 ± 0.10^ab^	0.70 ± 0.06^b^	4.08 ± 0.17^a^	3.09 ± 0.11^d^	2.33 ± 0.09^c^	3.30 ± 0.15^ab^	4.30 ± 0.21^a^
C18:0	0.22 ± 0.02^a^	0.23 ± 0.03^a^	0.24 ± 0.01^a^	0.17 ± 0.04^a^	0.27 ± 0.09^a^	0.98 ± 0.07^d^	0.76 ± 0.06^d^	1.92 ± 0.12^b^	1.48 ± 0.08^c^
C20:0	nd	0.14 ± 0.01^a^	0.13 ± 0.03^a^	0.10 ± 0.02^a^	0.21 ± 0.04^a^	0.06 ± 0.01^a^	0.13 ± 0.03^a^	1.57 ± 0.93^a^	1.26 ± 0.19^a^
C22:0	nd	1.00 ± 0.03^c^	1.87 ± 0.20^d^	0.30 ± 0.03^a^	2.70 ± 0.10^b^	0.96 ± 0.15^c^	0.25 ± 0.08^a^	nd	0.48 ± 0.09^c^
**∑SFA**	**28.51 ± 2.31^a^**	**29.25 ± 0.69^a^**	**30.68 ± 1.28^a^**	**24.28 ± 1.16^a^**	**29.34 ± 0.94^a^**	**28.00 ± 2.01^a^**	**28.67 ± 1.87^a^**	**32.65 ± 1.38^a^**	**30.02 ± 0.86^a^**
C16:1	1.33 ± 0.13^a^	0.25 ± 0.09^a^	1.99 ± 0.22^ab^	0.68 ± 0.08^a^	1.23 ± 0.31^a^	2.91 ± 0.26^c^	1.25 ± 0.15^a^	12.74 ± 0.48^b^	1.71 ± 0.20^ab^
C18:1n9t	4.54 ± 0.38^bc^	4.75 ± 0.55^b^	3.56 ± 0.31^bc^	0.26 ± 0.08^a^	1.99 ± 0.11^c^	4.18 ± 0.22^bc^	1.29 ± 0.09^ab^	2.83 ± 0.17^c^	3.19 ± 0.28^c^
C18:1n9c	32.88 ± 3.03^a^	35.80 ± 2.31^a^	33.81 ± 1.80^a^	36.76 ± 2.09^a^	37.08 ± 1.66^a^	29.08 ± 1.23^a^	30.21 ± 2.22^a^	26.64 ± 1.37^a^	34.54 ± 0.98^a^
C17:1	0.63 ± 0.06^a^	0.46 ± 0.07^a^	0.51 ± 0.10^a^	4.11 ± 0.26^c^	0.66 ± 0.09^a^	0.76 ± 0.09^a^	5.96 ± 0.12^b^	nd	1.00 ± 0.08^a^
C20:1n-9	0.98 ± 0.09^a^	0.17 ± 0.02^ab^	0.32 ± 0.03^ab^	0.98 ± 0.08^a^	nd	0.77 ± 0.05^a^	7.33 ± 0.17^b^	0.65 ± 0.07^a^	nd
C24:1	4.04 ± 0.13^f^	0.96 ± 0.08^a^	2.81 ± 0.07^e^	2.46 ± 0.09^d^	1.67 ± 0.01^c^	7.44 ± 0.12^b^	2.85 ± 0.09^e^	nd	nd
**∑MUFA**	**44.40 ± 3.01^a^**	**42.38 ± 2.12^a^**	**43.00 ± 1.89^a^**	**45.24 ± 1.22^a^**	**42.62 ± 2.60^a^**	**45.15 ± 2.44^a^**	**48.87 ± 4.01^a^**	**42.86 ± 1.83^a^**	**40.44 ± 1.11^a^**
C18:2n6t	3.07 ± 0.08^a^	2.45 ± 0.07^a^	2.89 ± 0.10^a^	4.76 ± 0.23^b^	2.61 ± 0.13^a^	2.58 ± 0.08^a^	5.17 ± 0.14^b^	3.77 ± 0.09^c^	2.17 ± 0.02^ab^
C18:2n6c	19.26 ± 1.49^a^	21.71 ± 2.08^a^	19.80 ± 0.59^a^	20.46 ± 1.44^a^	21.22 ± 1.61^a^	18.54 ± 0.89^a^	12.50 ± 0.99^a^	12.67 ± 1.05^a^	17.73 ± 2.06^a^
C20:2	1.72 ± 0.14^c^	0.26 ± 0.03^a^	0.97 ± 0.09^d^	2.17 ± 0.13^bc^	0.87 ± 0.06^d^	1.78 ± 0.08^c^	0.79 ± 0.05^d^	nd	2.30 ± 0.14^b^
C20:3n3	0.89 ± 0.07^b^	2.07 ± 0.14^a^	0.79 ± 0.06^b^	0.81 ± 0.04^b^	0.75 ± 0.07^b^	0.67 ± 0.03^b^	1.78 ± 0.11^a^	1.13 ± 0.08^b^	1.15 ± 0.09^b^
C22:5n-3	nd	0.94 ± 0.09^c^	0.56 ± 0.05^a^	0.48 ± 0.03^a^	0.97 ± 0.11^c^	nd	0.26 ± 0.02^a^	2.63 ± 0.09^b^	1.04 ± 0.12^c^
C22:2	0.60 ± 0.07^ac^	0.23 ± 0.01^a^	0.24 ± 0.04^a^	0.92 ± 0.13^ac^	0.58 ± 0.06^ac^	nd	0.84 ± 0.04^c^	2.57 ± 0.13^b^	1.42 ± 0.16^d^
C20:4n6	1.28 ± 0.10^c^	0.56 ± 0.06^a^	1.07 ± 0.07^c^	0.88 ± 0.08^ac^	1.05 ± 0.09^c^	1.00 ± 0.06^c^	1.07 ± 0.04^c^	1.72 ± 0.09^d^	3.73 ± 0.08^b^
C22:6n-3	0.28 ± 0.03^a^	0.13 ± 0.01^a^	nd	nd	nd	2.27 ± 0.12^b^	0.04 ± 0.01^a^	nd	nd
**∑PUFA**	**27.09 ± 0.45^a^**	**28.37 ± 2.15^a^**	**26.32 ± 1.34^a^**	**30.48 ± 0.99^a^**	**28.04 ± 1.78^a^**	**26.85 ± 0.98^a^**	**22.46 ± 3.68^a^**	**24.49 ± 1.85^a^**	**29.54 ± 1.05^a^**
∑n-3	1.17 ± 0.10^a^	3.15 ± 0.21^b^	1.35 ± 0.09^a^	1.29 ± 0.28^a^	1.72 ± 0.17^a^	2.94 ± 0.30^b^	2.08 ± 0.16^a^	3.76 ± 0.22^b^	2.19 ± 0.13^a^
∑n-6	23.60 ± 2.21^a^	24.73 ± 1.78^a^	23.76 ± 0.97^a^	26.10 ± 1.68^a^	24.88 ± 1.82^a^	22.12 ± 2.47^a^	18.74 ± 1.09^a^	18.16 ± 1.43^a^	23.63 ± 0.69^a^
**n-3/n-6**	**0.05 ± 0.02^a^**	**0.13 ± 0.08^a^**	**0.06 ± 0.01^a^**	**0.05 ± 0.01^a^**	**0.07 ± 0.02^a^**	**0.13 ± 0.05^a^**	**0.11 ± 0.07^a^**	**0.21 ± 0.09^a^**	**0.09 ± 0.01^a^**
**PUFA/SFA**	**0.95 ± 0.09^a^**	**0.97 ± 0.10^a^**	**0.86 ± 0.06^a^**	**1.26 ± 0.18^a^**	**0.96 ± 0.12^a^**	**0.96 ± 0.20^a^**	**0.78 ± 0.03^a^**	**0.75 ± 0.11^a^**	**0.98 ± 0.19^a^**

PFW: Plasma functionalized water, PFB: Plasma functionalized buffer, USPFW: Plasma functionalized water combined with ultrasound treatment, USPFB: Plasma functionalized buffer combined with ultrasound treatment, nd: not detected, SFA: saturated fatty acids, MUFA: monounsaturated fatty acids, PUFA: polyunsaturated fatty acids, Results are mean ± SEM. Different letters in the same row are significantly different (*p* < 0.05).

Furthermore, the availability and levels of C20:5n-3 (eicosapentaenoic acid: EPA) and C22:6n-3 (docosahexaenoic acid: DHA) are necessary for growth, development and promoting good health [58]. EPA was not detected in control samples but was induced in the range of 0.26–2.63% for treated samples, while DHA was enhanced to 2.27% after treatment from 0.28% for control samples. Treatment also led to insignificant increases in n-3/n-6 and PUFA/SFA ratio in the range of 0.05–0.21 and 0.75–1.26%, relative to control contents of 0.05 and 0.75%, respectively. The n-3 fatty acids are associated with the amelioration of cardiovascular disorders, suggesting the vital role of n-3/n-6 ratio as important biomedical index for fatty acids, and maintaining high PUF/SFA ratio in diets from the minimum recommended value of 0.45 can help prevent diseases [43], [58]. Thus, PFL decontamination (PFW and PFB) and their combination with ultrasound treatment (USPFW and USPFB) have the potential to improve the biomedical index and nutritional value of grass carp fatty acids and lipids.

### 3.6 Changes in textural profile analysis

Table 2 presents the changes in the textural profile analysis of grass carp during decontamination and the results indicated decreases in hardness and cohesiveness and slight increases in adhesiveness, springiness and chewiness for all treatment groups and voltage levels. Particularly, the hardness of grass carp was reduced to 515.65 g after treatment, relative to initial and control values of 594.48 and 584.30 g, respectively, while the cohesiveness was reduced to 0.65, relative to initial and control values of 0.071 and 0.070, respectively. On the other hand, adhesiveness, springiness and chewiness were slightly increased to −13.31 gs, 0.83 and 5.31, respectively, relative to initial values of −16.20 gs, 0.81 and 3.64, and control values of −16.13 gs, 0.79 and 3.75, respectively. However, the changes were only significant for hardness and chewiness, and the significance was only across the voltage levels and not the treatment group. It was also observed that combined treatments (USPFW and USPFB) presented higher hardness and springiness values when compared with individual PFW or PFB application, with USPFW producing the highest values.

**Table 2:** Effects of decontamination on the textural profile analysis and colour properties of grass carp.

Treatments	Applied Voltage (V)					
	0	30	40	50	60	70
***Hardness (g)***						
PFW	584.30 ± 14.61^aA^	535.30 ± 25.30 ^aA^	526.26 ± 24.72 ^aA^	523.69 ± 13.15 ^aA^	533.05 ± 14.91 ^aA^	515.65 ± 20.58 ^aA^
PFB	584.30 ± 14.61^aA^	527.33 ± 21.44 ^aA^	511.34 ± 9.33 ^aA^	524.54 ± 27.68 ^aA^	529.45 ± 19.88 ^aA^	523.50 ± 14.84 ^aA^
USPFW	584.30 ± 14.61^aA^	563.08 ± 7.44 ^aA^	558.02 ± 7.32 ^aA^	551.04 ± 9.01 ^aA^	535.78 ± 7.17 ^aA^	554.60 ± 13.64 ^aA^
USPFB	584.30 ± 14.61^aA^	550.41 ± 15.52 ^aA^	548.51 ± 18.72 ^aA^	544.03 ± 15.82 ^aA^	551.28 ± 22.61 ^aA^	531.19 ± 11.27 ^aA^

***Adhesiveness (gs)***						
PFW	−16.13 ± 0.45 ^aA^	−15.46 ± 1.09 ^aA^	−13.31 ± 1.44 ^aA^	−14.37 ± 0.60 ^aA^	−14.04 ± 0.26 ^aA^	−13.98 ± 0.54 ^aA^
PFB	−16.13 ± 0.45 ^aA^	−14.89 ± 1.22 ^aA^	−15.05 ± 0.19 ^aA^	−14.08 ± 1.09 ^aA^	−13.81 ± 0.54 ^aA^	−14.05 ± 0.86 ^aA^
USPFW	−16.13 ± 0.45 ^aA^	−15.92 ± 0.53 ^aA^	−14.32 ± 0.34 ^aA^	−15.07 ± 0.10 ^aA^	−14.39 ± 0.41 ^aA^	−14.50 ± 1.14 ^aA^
USPFB	−16.13 ± 0.45 ^aA^	−15.46 ± 0.59 ^aA^	−14.61 ± 0.74 ^aA^	−14.72 ± 0.67 ^aA^	−14.07 ± 0.08 ^aA^	−14.54 ± 0.66 ^aA^

***Springiness***						
PFW	0.79 ± 0.10 ^aA^	0.80 ± 0.05 ^aA^	0.78 ± 0.05 ^aA^	0.81 ± 0.02 ^aA^	0.82 ± 0.03 ^aA^	0.82 ± 0.06 ^aA^
PFB	0.79 ± 0.10 ^aA^	0.79 ± 0.01 ^aA^	0.80 ± 0.07 ^aA^	0.81 ± 0.07 ^aA^	0.82 ± 0.04 ^aA^	0.80 ± 0.04 ^aA^
USPFW	0.79 ± 0.10 ^aA^	0.80 ± 0.05 ^aA^	0.79 ± 0.12 ^aA^	0.83 ± 0.07 ^aA^	0.83 ± 0.04 ^aA^	0.79 ± 0.06 ^aA^
USPFB	0.79 ± 0.10 ^aA^	0.82 ± 0.06 ^aA^	0.80 ± 0.05 ^aA^	0.78 ± 0.05 ^aA^	0.79 ± 0.14 ^aA^	0.81 ± 0.03 ^aA^

***Cohesiveness***						
PFW	0.070 ± 0.0075 ^aA^	0.068 ± 0.0020 ^aA^	0.067 ± 0.0085 ^aA^	0.068 ± 0.0080 ^aA^	0.068 ± 0.0025 ^aA^	0.067 ± 0.0035 ^aA^
PFB	0.070 ± 0.0075 ^aA^	0.069 ± 0.0035 ^aA^	0.067 ± 0.0065 ^aA^	0.069 ± 0.0050 ^aA^	0.066 ± 0.0040 ^aA^	0.066 ± 0.0045 ^aA^
USPFW	0.070 ± 0.0075 ^aA^	0.065 ± 0.0025 ^aA^	0.067 ± 0.0055 ^aA^	0.065 ± 0.0050 ^aA^	0.065 ± 0.0060 ^aA^	0.066 ± 0.0060 ^aA^
USPFB	0.070 ± 0.0075 ^aA^	0.066 ± 0.0035 ^aA^	0.065 ± 0.0055 ^aA^	0.066 ± 0.0060 ^aA^	0.066 ± 0.0050 ^aA^	0.065 ± 0.0040 ^aA^

***Chewiness***						
PFW	3.750 ± 0.049 ^aA^	3.731 ± 0.230 ^aA^	4.047 ± 0.258 ^aA^	4.668 ± 1.052 ^aA^	5.091 ± 0.959 ^aA^	5.309 ± 0.112 ^aA^
PFB	3.750 ± 0.049 ^aA^	3.944 ± 0.106 ^aA^	4.612 ± 0.402 ^aA^	4.774 ± 0.694 ^aA^	4.425 ± 0.555 ^aA^	5.252 ± 0.688 ^aA^
USPFW	3.750 ± 0.049 ^aA^	3.926 ± 0.197 ^aA^	4.003 ± 0.214 ^aA^	4.191 ± 0.327 ^aA^	5.006 ± 0.652 ^aA^	4.894 ± 0.533 ^aA^
USPFB	3.750 ± 0.049 ^aA^	3.309 ± 0.639 ^aA^	4.122 ± 0.337 ^aA^	5.109 ± 0.427 ^aA^	4.208 ± 0.378 ^aA^	4.875 ± 0.225 ^aA^

***Net colour difference***						
PFW	1.80 ± 0.20^aA^	2.10 ± 0.10^aA^	2.87 ± 0.16^bcA^	3.63 ± 0.17^cA^	4.30 ± 0.10^bB^	4.25 ± 0.14^bcA^
PFB	1.80 ± 0.20^aA^	4.12 ± 0.11^cC^	4.71 ± 0.12^cC^	6.24 ± 0.07^dC^	7.21 ± 0.15^eA^	8.39 ± 0.17^bD^
USPFW	1.80 ± 0.20^aA^	3.89 ± 0.12^cC^	4.77 ± 0.16^dC^	4.13 ± 0.09^cA^	5.37 ± 0.08^dC^	6.29 ± 0.11^bC^
USPFB	1.80 ± 0.20^aA^	5.66 ± 0.08^cB^	6.69 ± 0.14 ^dB^	7.64 ± 0.16^eB^	7.29 ± 0.05d^eA^	9.09 ± 0.19^bB^

***Whiteness index***						
PFW	32.61 ± 0.98^aA^	36.08 ± 0.12^abB^	39.68 ± 1.55^bAB^	41.17 ± 1.17^bAB^	40.45 ± 1.45^bB^	41.70 ± 1.30^bA^
PFB	32.61 ± 0.98^aA^	34.80 ± 0.32^abAB^	35.80 ± 1.20^abAB^	36.98 ± 1.14^abB^	37.55 ± 1.55^abAB^	38.92 ± 1.02^bA^
USPFW	32.61 ± 0.98^aA^	40.75 ± 1.05^bB^	40.50 ± 1.50^bA^	42.62 ± 1.94^bA^	44.08 ± 1.22^bB^	42.35 ± 2.05^bA^
USPFB	32.61 ± 0.98^aA^	35.95 ± 0.95^abB^	34.98 ± 1.24^abB^	34.41 ± 1.92^abB^	37.40 ± 0.72^abAB^	39.80 ± 0.80^bA^

PFW = Plasma functionalized water, PFB = Plasma functionalized buffer. USPFW = Plasma functionalized water combined with ultrasound treatment. USPFB = Plasma functionalized buffer combined with ultrasound treatment. Error bars represent standard error of measurement. Different lowercase letters in the same row and different uppercase letters in the same column are significantly different (*p* < 0.05).

The textural profile of muscle foods is usually associated with species, muscle fibre density, fat and protein contents and water-holding capacity, and oxidation-induced water loss can reduce the water-holding capacity by damaging the membrane structural integrity of muscles fibres [59], [60]. The hardness often substituted by firmness relates to the peak force during the first compression cycle or first bite, cohesiveness measures the rate, at which the muscle disintegrate during mastication, adhesiveness corresponds to the work required to overcome the attractive forces between muscle surface and other surfaces that it is in contact with, springiness relates to the recovery height of the muscle between the end of the first bite and start of the second bite, while chewiness measures the energy required for mastication [60], [61]. The decreases in hardness and cohesiveness were possibly due to the accelerated oxidation of proteins by the increasing concentration of RONS generated in PFL. A decrease in the hardness and comparable TPA of beef, black sea bream and Nile tilapia have also been reported during the thawing process with PFW, immersing in OFW and OFW combined with tea polyphenol [5], [12], [60].

The reason for the hardness and springiness values of samples immersed in PFL and simultaneously treated by ultrasound being higher than individual application was not well understood. However, comparable firmness values between untreated chicken muscle and samples immersed in PFW combined with ultrasound, and untreated salmon and samples immersed in EFW combined with UV-C radiation and ultrasound have been reported [10], [11]. Aggregation and denaturation of myofibrillar protein have been linked to tissue hardening of fish [60]. Ultrasound treatment is known to generate compression and mechanical shockwaves, which may enhance grass carp myofibrillar protein aggregation and denaturation, responsible for higher firmness values of samples from simultaneous treatment by PFL and ultrasound in the current study.

### 3.7 Changes in colour properties

Fig. 4b shows the visual observation of colour changes in grass carp during treatment and the changes were expressed as NCD and WI (Table 2), indicating that PFL treatment markedly influenced the colour parameters. A significant increase (p < 0.05) in NCD was observed for immersing the samples in PFW and PFB and the values increased with increasing applied voltages, ranging from 2.10 to 4.25 for PFW and 4.12 – 8.39 for PFB, as compared with the control value of 1.80, and the combined treatment with ultrasound further increased the NCD values, reaching 6.29 for USPFW and 9.09 for USPFB. NCD values for samples in PFB were higher than in PFW and for USPFW. The WI in PFL and their combination with ultrasound treatment also increased significantly (p < 0.05) with increasing applied voltages as samples immersed in DW presented WI value of 32.61 as compared with the highest values of 41.70 and 38.92 for samples in PFW and PFB, respectively, while combined treatment with ultrasound presented WI values in the range of 40.50–44.08 and 34.41–39.80 for USPFW and USPFB, respectively, with USPFW samples having the highest values.

The colour of fish has been reported to be variably influenced by the amount of bound water, pigment concentrations and muscle structure characteristics, and is considered as an important factor for determining the acceptance of fish products [12], [62]. The distinct colour change and alternation in WI especially for the high voltage application might be attributed to the bleaching and oxidation of carotenoid, haemoglobin and myoglobin pigments by RONS generated in PFL, in addition to enzymatic and non-enzymatic reactions, which results in the disorganisation of myofibrils and degradation of myofibrillar proteins [62]. The pattern of variation in the current study have been reported in the literature, where the brightness indicators (WI) of salmon and Bombay duck fillets immersed in EFW and combined with ultrasound treatment, UV-C and botanic bio-preservatives were higher than the control samples and those immersed in EFW alone [11], [62]. In contrast, the WI of black sea bream immersed in OFW in combination with tea polyphenol were lower than the control samples and samples immersed in OFW alone [12].

### 3.8 Changes in protein structure

**Fig. 5:**
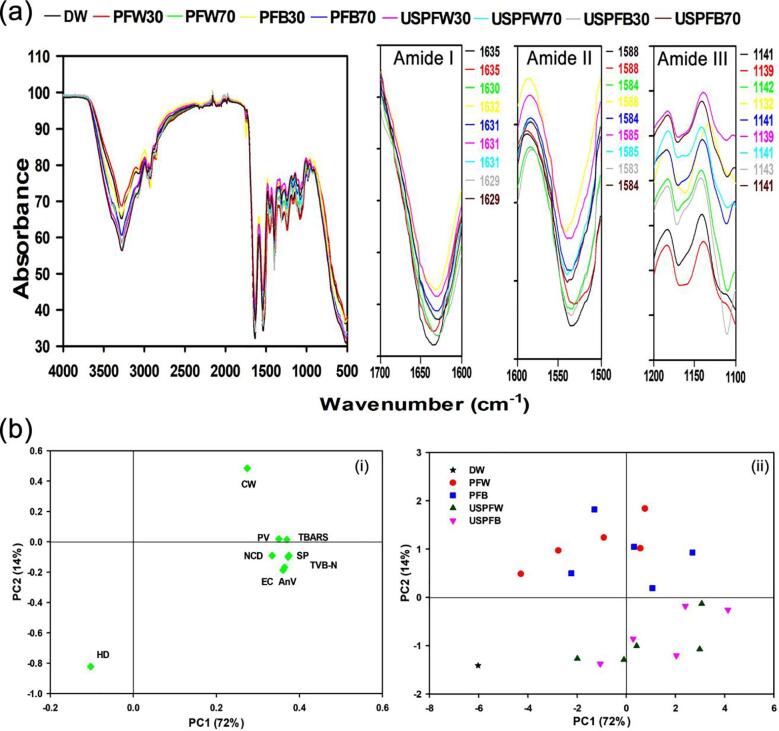
(a) Changes in protein structure of grass carp during decontamination and (b) Principal component analysis of (i) loading variable plot and (ii) score plot showing variations among decontamination methods (CW: chewiness, PV: peroxide value, TBARS: thiobarbituric acid reactive substances, NCD: net colour change, TVB-N: total volatile basic nitrogen, AnV: *para*-anisidine value, SP: *S*. *putrefaciens* log reduction, EC: *E. coli* log reduction).

The FTIR spectra of grass carp muscle during decontamination is presented in Fig. 5a and only data for 30 and 70 V were reported for improved visibility and interpretation since variations among voltages was minimal. The spectra exhibited similar trends and features for all samples and the peak intensity decreased with treatment, indicating protein denaturation and unfolding from the actions of RONS in PFL and cavitation-induced shear actions of ultrasound treatment [63], [64], [65]. Similar results have been reported for Atlantic salmon treated with acidic electrolyzed water [22]. FTIR is typically used to evaluate the secondary structure of proteins and the spectra region of 110–1700 cm^−1^ provides valuable information on protein polypeptide conformation [66].

Furthermore, the maximum absorbance in the amide I band was at 1635 cm^−1^ for fresh grass carp and this shifted to 1630 and 1629 cm^−1^, respectively, for individual and combination treatments, indicating a slight transition as a result of treatment. The amide I band located between 1600 and 1700 cm^−1^ typically indicates C = O stretching vibrations, C–C-N deformation, C-N stretch, in-plane bending, and is generally referred to as the fingerprint for protein secondary structure [66]. Spectral changes in this region have been associated with soy protein hydrolysates and salmon conformational changes as a result of ultrasound and electrolyzed water processing [22], [66]. Amide II (1500–1600 cm^−1^) and amide III (1100–1600 cm^−1^) bands corresponding to N–H bending and protein side chains chemical groups, respectively, are used to further substantiate analysis from amide I due to weak absorption of proteins at these bands, and data showed a maximum shift from 1588 to 1583 cm^−1^, and 1141 to 1132 cm^−1^, respectively. Overall, results showed a slight transition in the secondary structure of the protein from treatment and such changes were accompanied by improved biological properties of proteins in food model systems [66].

### 3.9 Multivariate data analysis

PCA was performed to present a comprehensive insight into the significant variations during decontamination, and the first two principal components with eigenvalues greater than 1 and considered significant from the Kaiser criterion [67] were extracted and are presented in Fig. 5b. According to the loading plot, the first principal component (PC1) correlated positively with microbial reduction, AnV, TBARS, PV, TVB-N, and NCD, accounting for 72% of the total variance, while the second principal component (PC2) negatively correlated with hardness values, accounting for 14% of the total variance. Together, PC1 and PC2 explained 86% of the total variance. Fig. 5b(ii) presents the comprehensive score plot that compares samples under different decontamination methods and data clustering together in a score plot typically suggests high similarities with respect to the parameters under investigation. In this case, individual treatments (PFW and PFB) were clustered together and combined treatments (USPFW and USPFB) were scattered together, suggesting similar effects of decontamination from each clustered group. In addition, PFW and PFB at 60–70 V showed positive PC1 to suggest high values of TBARS, PV and chewiness. USPFW and USPFB at 50–70 V showed positive PC1 scores, suggesting their higher microbial reduction and NCD, TVB-N and AnV values. The divisions further indicated the effect of decontamination on the properties of grass carp, for instance, USPFW and USPFB at 30–40 V and DW showed negative PC1 scores, indicating a close relationship hardness of grass carp and these treatments.

## 4 Conclusions

The current study showed that PFL including PFW and PFB applied individually or in combination with ultrasound technology for grass carp decontamination had significant effects on pathogenic bacteria reduction, in particular, the combination of PFL and ultrasound treatment (USPFW and USPFB) showed improved inactivation of *E. coli* and *S*. *putrefaciens* inoculated on raw grass carp fillets, with potential synergy demonstrated for PFW and ultrasound. However, the combined treatments induced increases in lipid oxidation and protein degradation as compared with individual applications, but the values were generally below the acceptable limits, while the reduction in hardness was better retarded by the combined treatments. Besides, the treatment also led to improved biomedical index and nutritional value of grass carp fatty acids and lipids, denaturation and structural unfolding of proteins, which is typically accompanied by improved functional properties, and samples from combined treatment exhibited distinct colour change and increased brightness of grass carp. An implication of the current study is the improved utilization of grass carp due to improved safety, possible enhancement of biomedical index and nutritional value of the fatty acids and lipids, enhanced protein biological activity, and the evolution of broad-spectrum sanitisers that can be used individually or in combination with ultrasound for improved safety and quality of seafood products. Nevertheless, given the complexity of the process and the relative marginal effect, further research is required, especially on the optimization of treatment conditions for enhanced impact and minimal changes in quality parameters.

## CRediT authorship contribution statement

**Okon Johnson Esua:** Writing – original draft, Formal analysis, Investigation. **Jun-Hu Cheng:** Validation, Funding acquisition, Resources. **Da-Wen Sun:** Supervision, Funding acquisition, Resources, Writing - review & editing.

## Declaration of Competing Interest

The authors declare that they have no known competing financial interests or personal relationships that could have appeared to influence the work reported in this paper.
